# Supplementary material for: A randomised controlled trial to reduce highest priority critically important antimicrobial prescription in companion animals
Source: Nat Commun. 2021 Mar 11;12:1593. doi: 10.1038/s41467-021-21864-3 (PMC7952375; doi:10.1038/s41467-021-21864-3)
Supplement: Supplementary file 1 — Supplementary Information [file 41467_2021_21864_MOESM1_ESM.pdf]

Supplementary Information

Supplementary Note 1: Benchmarking intervention letter

Delivered to low and high intervention group practices, alerting them to their above average use of highest priority critically important antimicrobials.

Dear Practice Team,

Antimicrobial resistance has become a significant public health issue and there is now increasing interest in the use of antibacterials in companion animals, both because of the importance of companion animals in human health and because of the recognition of resistant bacteria in companion animal practice.

It is vitally important that the veterinary profession embraces the responsible use of antibacterials in order to:

- Minimize selection of resistant veterinary pathogens, and therefore safeguard animal health
- Minimize possible transfer of resistance to human pathogens
- Retain the right to prescribe certain antibacterials that are important in human medicine, e.g. the fluoroquinolones and third-generation cephalosporins

Based on data submitted to SAVSNET between August 2018 and January 2019 inclusive, this practice has been identified as an above average prescriber of highest priority critically important antibiotics (HPCIA – which include fluoroquinolones and third-generation cephalosporins) within CVS via the SAVSNET portal. As a group, we are keen to work with our vets to help them ensure appropriate use of all antibiotics, but especially these HPCIAs. We would now like to encourage your team to review the use of antibiotics at the practice.

There are many tools available to assist in the review, which include:

- Your SAVSNET portal
- CVS AMR guidelines (included)
- BSAVA PROTECT ME guidelines
- We also encourage you to view the webinar (web link)

Please note, we will not be dictating a prescribing policy; your vets will still have freedom to prescribe the treatments they feel are most appropriate to their patients.

It is important as a group we can understand the impact of such an audit. Therefore, with SAVSNET support, we will be following your future antibiotic use and comparing it to other practices that similarly use high levels of antibiotics, but who have not been asked to review their use. This follow up period will last approximately six months from April to September 2019 inclusive. During this period you will be able to benchmark your antibiotic use through the SAVSNET portal. With University of Liverpool and SAVSNET, we may look to publish this work; if we do, your participation would be entirely anonymous and you will see the publication before it is submitted.

Your participation in this audit is of course voluntary and if you decide not to take part, it will have no impact on how you are viewed within the group.

Kind regards,

Director of Clinical Services

Supplementary Note 2: Example antimicrobial prescription benchmarking portal screenshot

Example page of the anonymised benchmarking portal available to all practices participating in SAVSNET. Each practice holds a unique login to this portal, enabling further understanding of their own data compared to their peers.

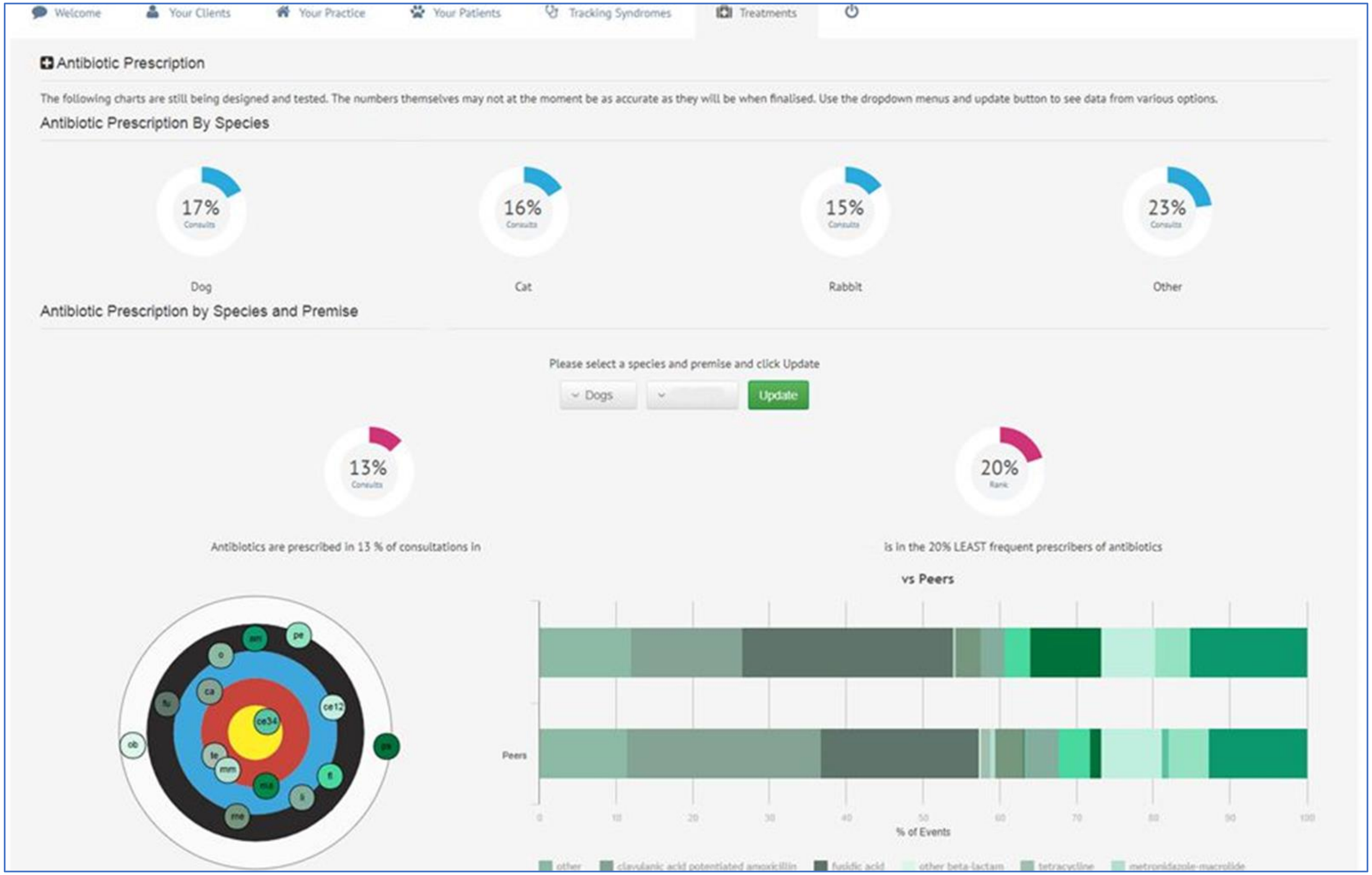

Supplementary Note 3: Exemplar in-depth antimicrobial prescription benchmarking report

Provided to all high intervention group practices, providing an increased level of detail in comparison to that available to the low intervention and control group practices. Mocked up data provided below.

The Small Animal Veterinary Surveillance Network Antibiotic Prescription Tracker

Practice name: SAVSNET Practice

Welcome to your first mySavsnet AMR antibiotic prescription tracker report! Below you will find a summary of the frequency and variety with which your practice prescribes antibiotics. This has been anonymously benchmarked against veterinary practices which currently take part in SAVSNET (www.savsnet.co.uk).

As I am sure you are aware, antimicrobial resistance (AMR) is a global health threat of increasing concern to both animals and humans. The recent global Review on Antimicrobial Resistance (https://amr-review.org/) has estimated that currently an estimated 700,000 people per year die as a consequence of AMR, this could rise to 10,000,000 people dying per year by 2050. It is not known what the impact on animal welfare will be.

One of the key things we can all do to help slow the rate of AMR development is to practice good antibiotic stewardship. We hope this report will help you reflect on your / your practice's own antibiotic prescription patterns, helping you to understand how your prescription compares to others. Whatever you conclude from this report, we would also recommend taking the time to read about the BSAVA PROTECT ME Scheme, a project with the aim of helping veterinary practices develop their own responsible antibiotic prescription policies.

In the meantime, we hope you enjoy taking a fresh look at your antibiotic prescription! If you have any questions please email savsnet@liv.ac.uk.

Vital Statistics

Parameters	Dog	Cat
Consultation analysis dates	1 October 2018 — 25 March 2019	
Total recorded consultations	4532	2035
Number of submitted consultations[1]	Largest 20%	Largest 30%
% consultations prescribed an antibiotic	20.7%	14.6%
% consultations prescribed a systemic antibiotic	12.0%	14.6%
% consultations prescribed a topical antibiotic	8.7%	3.1%
% consultations prescribed a HPCIA (see over)	0.6%	5.0%
% consultations prescribed a systemic HPCIA	0.3%	4.8%
Most commonly prescribed antibiotic class	Amoxiclav	3rd gen. cephalosporin

1 Compared to other data-submitting veterinary practices, the relative number of consultations your practice submitted to this project. Smallest = smallest number of submitted consultations, Largest = Largest number of submitted consultations.

If you would like to known more about SAVSNET, please email: savsnet@liv.ac.uk

The Small Animal Veterinary Surveillance Network Antibiotic Prescription Tracker

How frequently do you prescribe antibiotics?

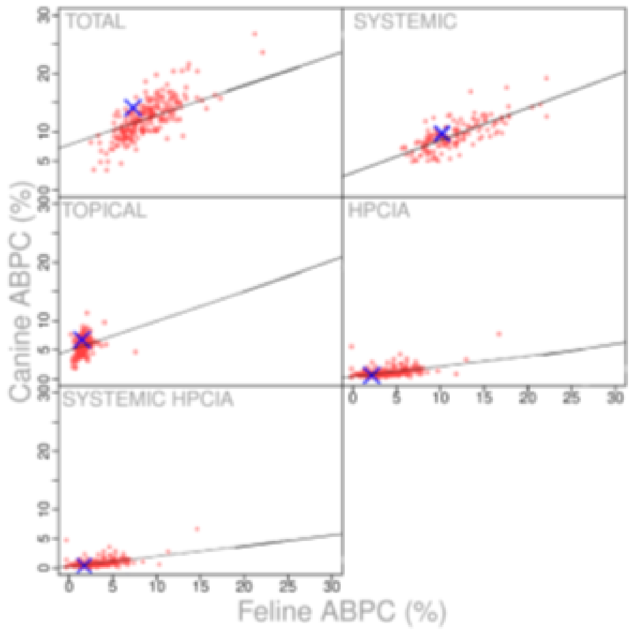

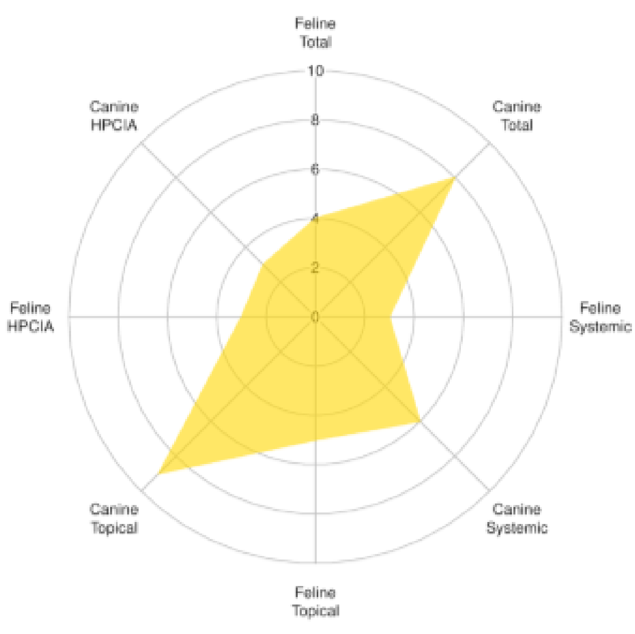

The graph above left displays the percentage of canine and feline consultations where at least one antibiotic was prescribed (ABPC) by your practice (blue cross), compared with every other practice that has submitted data (red points). We have summarised antibiotics prescribed based on route of administration (systemic, topical) and 'HPCIA', otherwise known as 'highest priority critically important antibiotics'. If you would like to know more about HPCIAs please refer to the box below.

The radar plot above right displays your antibiotic prescription frequency for dogs and cats for each of the five antibiotic categories noted above. You / your practice has been placed into a decile for each category where, for example, 10 = you are in the 10% highest frequency antibiotic prescribing veterinary practices for that category.

*What are HPCIAs?

Highest Priority Critically Important Antibiotics (HPCIA) refer to a group of antibiotic classes considered by the World Health Organisation to be of greatest importance to preserving human health. It is recommended that these classes are prescribed prudently in both people and veterinary species. Of particular relevance to companion animals, this list includes 3rd and 4th generation cephalosporins and fluoroquinolones. More information on HPCIAs can be seen here.

The Small Animal Veterinary Surveillance Network Antibiotic Prescription Tracker

Which antibiotics do you prescribe?

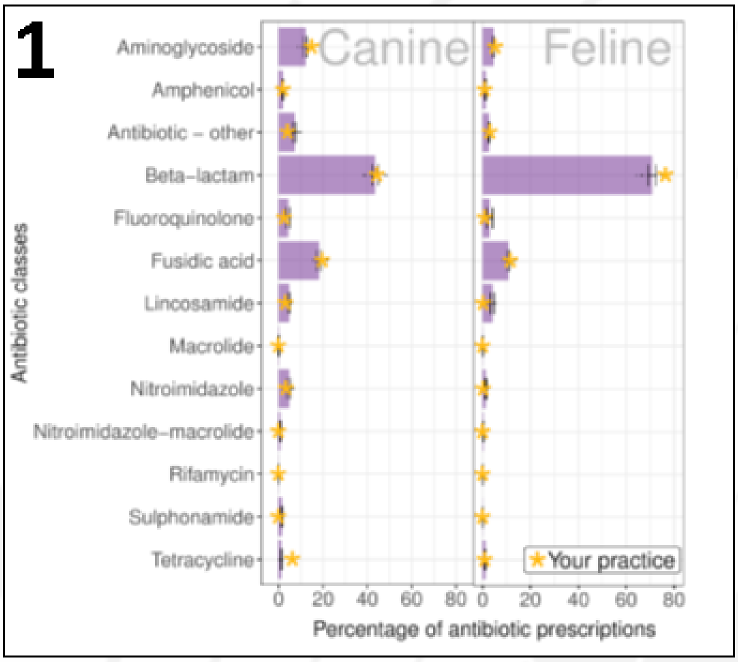

The graph to the left (labelled 1) displays your relative frequency of prescription of particular broad classes of antibiotic (yellow asterisk) as a percentage of the total number of antibiotic prescriptions you provided to us, compared against the whole population of practices that have submitted data to SAVSNET (purple bars). 95% confidence intervals are included, as is the inter-quartile range (1st-3rd, dotted line).

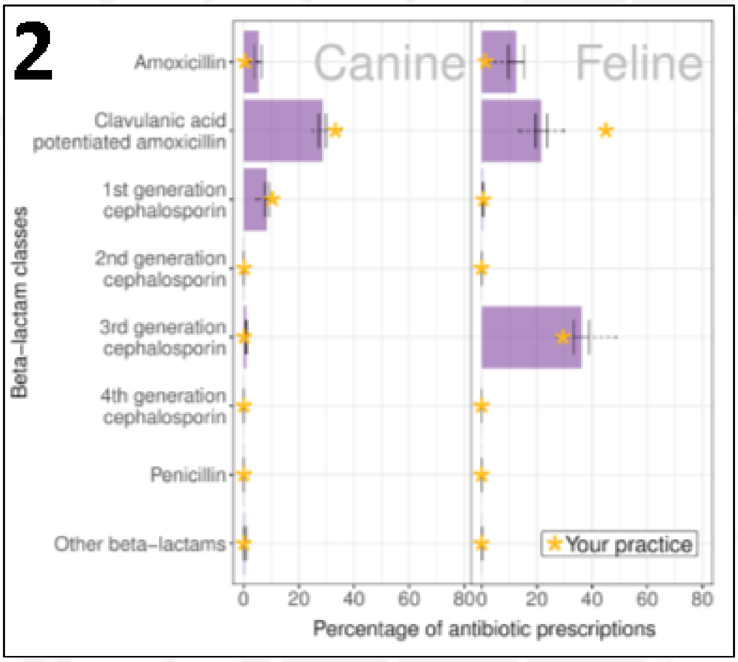

The graph to the left (labelled 2) displays a breakdown of the beta-lactam class into further sub-classes. Again you can see your relative practice's prescribing tendencies (yellow asterisk) compared against other data submitting veterinary practices. Of note, 3rd and 4th generation cephalosporins are classed as HPCIAs. To learn more about antibiotic categorisation click here.

If you would like to known more about SAVSNET, please email: savsnet@liv.ac.uk

What about different clinical presentations?

Considering particular clinical presentations, we used the 'main presenting complaint' (MPC) as recorded by the SAVSNET window to focus on six broad presentations more commonly associated with antibiotic prescription. The total number of consultations recorded by MPC analysed in this report is given in the table below.

Main presenting complaint	Dog	Cat
Gastroenteric	234	87
Respiratory	101	54
Pruritus	454	123
Trauma	432	365
Other unwell	686	576
Post-operative	561	234

The graphs below display antibiotic prescription frequency in dogs and cats for six broad clinical presentations, as indicated on the data you submitted, across five antibiotic administration and classification categories. You / your practice has been placed into a decile for each category where 10 = you are in the 10% highest frequency antibiotic prescribing veterinary practices for that category.

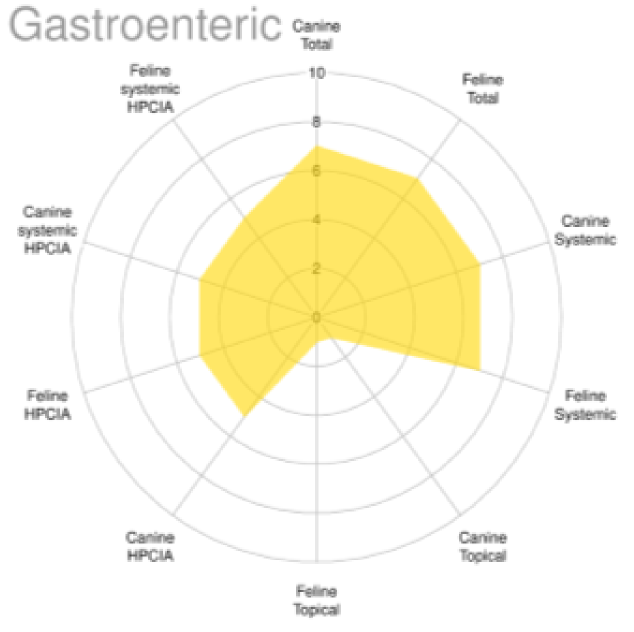

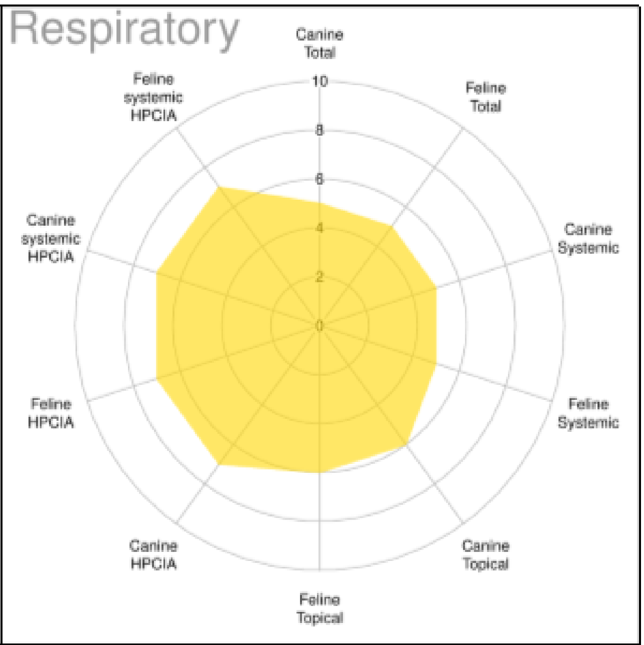

The Small Animal Veterinary Surveillance Network Antibiotic Prescription Tracker

What about different clinical presentations?

The graphs below display antibiotic prescription frequency in dogs and cats for six broad clinical presentations, as indicated on the data you submitted, across five antibiotic administration and classification categories. You / your practice has been placed into a decile for each category where 10 = you are in the 10% highest frequency antibiotic prescribing veterinary practices for that category.

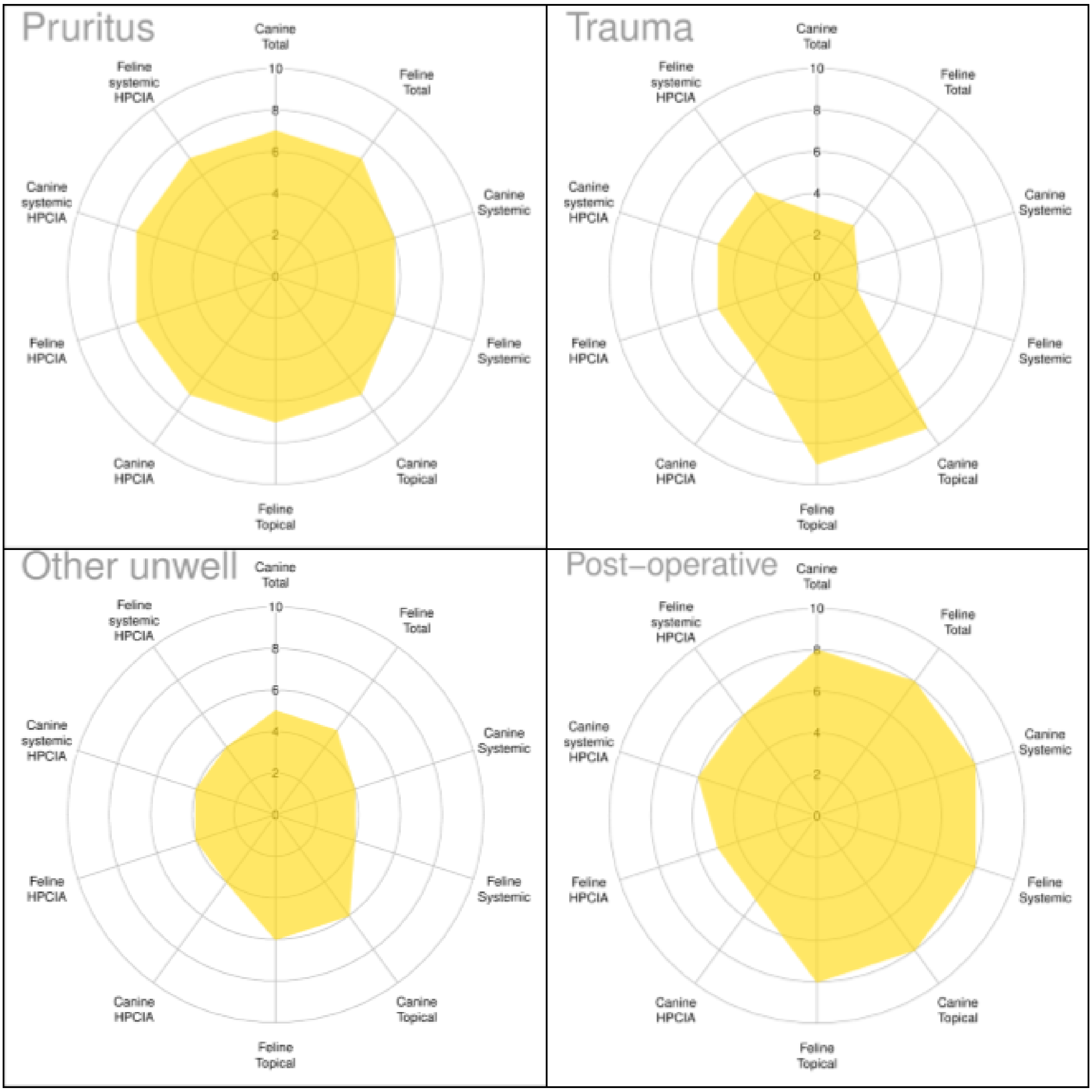

The Small Animal Veterinary Surveillance Network Antibiotic Prescription Tracker

Interpretation/notes

SAVSNET Practice submitted data in an electronic format, this practice already participating in the SAVSNET project. Our primary aim is to present your data so you can draw your own conclusions — as such we will not comment on your benchmarking statistics directly. However, from an interpretation perspective we note that this practice has submitted a large volume of consultations, providing relatively high confidence that our findings are representative of your practice's normal antibiotic prescribing habits.

We would also like to take the opportunity to thank you for taking the time to submit data to SAVSNET project! We would particularly appreciate it if you were able to give a few moments of your time to provide feedback on this report and the wider SAVSNET project. We are trying to develop such auditing/benchmarking into a regular feature of practice—hence, your thoughts at this stage are crucial for the future development of this project. We would also like to remind you of the existing antibiotic prescription benchmarking page on your personalised practice portal.

Feedback

If you would like to known more about SAVSNET, please email: savsnet@liv.ac.uk

Supplementary Note 4: Human factors practice review checklists

List of questions asked during a human factors framework review of high intervention group practice's culture and approach to antimicrobial prescription. There were three checklists: initial review, a further checklist for subsequent follow-up meetings, and a final study conclusion checklist. These questions were delivered by each hub clinical lead verbally within flow of conversation; as such it is unlikely these questions were delivered verbatim.

Initial review

1. Number of vets in practice / any recent staff changes
2. Does the team feel they can ask for each other's help with cases?
3. What is the team's view on responsible use of antibiotics?
4. Any patient factors that may contribute to prescribing without work-up?
5. Any owner factors that may contribute to prescribing without work-up?
6. Any workload or staffing issues that may prevent work-up?
7. Are the correct drugs, equipment, supplies and reference materials available and working properly?
8. Are there any characteristics about the drugs, equipment, disposables and reference material that are unhelpful?
9. Are there any improvements to be made in infection control?
10. Are there any issues with skill or knowledge?
11. Do local policies, guidelines, protocols or checklists help or hinder?
12. Are clinical notes sufficient to follow cases?
13. Practice concerns in implementing changes.
14. Actions to be taken to address issues identified above.
15. Date of practice meeting to review practice AMR policy/data
16. Agreed follow-up method

Follow-up review

1. Has the practice accessed the webinars?
2. Has the practice reviewed AMR policies?
3. Has the practice reviewed infection control guidance?
4. Has the practice reviewed previous cases?
5. Has the practice reviewed SAVSNET reports/data?
6. Outcome of the practice meeting
7. Revisit of initial review questions (detailed above) – any new information?
8. Any practice concerns?

Final review

1. Has the practice found the study a valuable exercise?
2. Does the practice plan to continue the audit after the study is over?
3. What tools did the practice find useful?
4. If training undertaken, what was completed?
5. If access to equipment/materials was improved, what was done?
6. Any feedback or requests from the practice?

Supplementary Table 1: Orthogonal polynomial contrast coding values utilised for construction of six mixed effects panel regression models, modelling the percentage of canine and feline highest priority critically important antimicrobial agent (HPCIA) antimicrobial agent prescription in practices belonging to control, light intervention or heavy intervention groups. Models incorporate practice as a random effect and the ordinal variable month as a fixed effect.

x	x^2	x^3	x^4	x^5	x^6	x^7	x^8	x^9	x^{10}	x^{11}	x^{12}	x^{13}	x^{14}	x^{15}
-0.41	0.46	-0.45	0.40	-0.32	0.23	-0.16	0.10	-0.05	0.03	-0.01	0.005	-0.002	0.0004	-0.0001
-0.35	0.28	-0.09	-0.13	0.32	-0.42	0.43	-0.37	0.27	-0.17	0.10	-0.05	0.02	-0.01	0.001
-0.30	0.12	0.14	-0.32	0.32	-0.14	-0.12	0.33	-0.42	0.40	-0.30	0.18	-0.09	0.03	-0.01
-0.24	-0.01	0.27	-0.29	0.07	0.21	-0.34	0.22	0.06	0.32	0.43	-0.38	0.25	-0.12	0.04
-0.19	-0.11	0.30	-0.15	-0.17	0.31	-0.13	-0.20	0.34	-0.17	-0.16	0.40	-0.42	0.28	-0.11
-0.14	-0.20	0.26	0.03	-0.29	0.16	0.19	-0.30	0.03	0.30	-0.29	-0.05	0.39	-0.43	0.24
-0.08	-0.25	0.18	0.19	-0.26	-0.09	0.30	-0.04	-0.30	0.18	0.23	-0.33	-0.05	0.43	-0.40
-0.03	-0.28	0.06	0.28	-0.10	-0.27	0.14	0.26	-0.19	-0.25	0.24	0.22	-0.32	-0.19	0.52
0.03	-0.28	-0.06	0.28	0.10	-0.27	0.14	0.26	0.19	-0.25	-0.24	0.22	0.32	-0.19	-0.52
0.08	-0.25	-0.18	0.19	0.26	-0.09	-0.30	-0.04	0.30	0.18	-0.23	-0.33	0.05	0.43	0.40
0.14	-0.20	-0.26	0.03	0.29	-.16	-0.19	-0.30	-0.03	0.30	0.29	-0.05	-0.39	-0.43	-0.24
0.19	-0.11	-0.30	-0.15	0.17	0.31	0.13	-0.20	-0.34	-0.17	0.16	0.40	0.42	0.28	0.11
0.24	-0.01	-0.27	-0.29	-0.07	0.21	0.34	0.22	-0.06	-0.32	-0.43	-0.38	-0.25	-0.12	-0.04
0.30	0.12	-0.14	-0.32	-0.32	-0.14	0.12	0.33	0.42	0.40	0.30	0.18	0.09	0.03	0.01
0.35	0.28	0.09	-0.13	-0.32	-0.42	-0.43	-0.37	-0.27	-0.17	-0.10	-0.05	-0.02	-0.01	-0.001
0.41	0.46	0.45	0.40	0.32	0.23	0.16	0.10	0.05	0.03	0.01	0.005	0.002	0.004	0.0001

Supplementary Table 2: Canine findings from a series of mixed effects panel regression models, measuring intervention group variation pre- and post-intervention across a range of antimicrobial prescription categories, measured as a percentage of total consultations.

Antimicrobial group	Random effects		Intervention stage	Intervention group	Fixed effects			Model fit	
	Category	Var [a] (SD [b])			Estimate	SE [c]	*P*	Chi-sq. (*P*)	R-sq.
**HPCIA [d]**	Practice	0.10 (0.32)	Pre-intervention	Control (intercept)	0.80	0.09	-	12.59	0.10
	Idiosyncratic	0.08 (0.32)	Post-intervention	Control	-0.01	0.09	0.88	(0.028)	
			Pre-intervention	Heavy	0.01	0.13	0.97		
			Post-intervention	Heavy	-0.27	0.13	0.04		
			Pre-intervention	Light	-0.12	0.13	0.36		
			Post-intervention	Light	-0.04	0.13	0.78		
**Total**	Practice	4.04 (2.01)	Pre-intervention	Control (intercept)	17.96	0.58	-	22.13	0.16
	Idiosyncratic	2.72 (1.65)	Post-intervention	Control	0.60	0.52	0.13	(<0.001)	
			Pre-intervention	Heavy	-0.39	0.82	0.64		
			Post-intervention	Heavy	-2.98	0.82	0.0002		
			Pre-intervention	Light	-0.04	0.82	0.96		
			Post-intervention	Light	-1.07	0.82	0.14		
**Systemic**	Practice	3.13 (1.77)	Pre-intervention	Control (intercept)	10.76	0.51	-	28.05	0.20
	Idiosyncratic	2.12 (1.46)	Post-intervention	Control	0.55	0.46	0.14	(<0.001)	
			Pre-intervention	Heavy	-0.18	0.72	0.81		
			Post-intervention	Heavy	-2.77	0.72	0.0001		
			Pre-intervention	Light	0.00	0.72	1.00		
			Post-intervention	Light	-1.16	0.72	0.11		
**Topical**	Practice	1.12 (1.06)	Pre-intervention	Control (intercept)	7.87	0.30	-	2.33	0.02
	Idiosyncratic	0.66 (0.81)	Post-intervention	Control	0.11	0.26	0.66	(0.803)	
			Pre-intervention	Heavy	-0.31	0.42	0.46		
			Post-intervention	Heavy	-0.57	0.42	0.18		
			Pre-intervention	Light	0.03	0.42	0.94		
			Post-intervention	Light	-0.05	0.42	0.91		
**Systemic HPCIA**	Practice	0.06 (0.25)	Pre-intervention	Control (intercept)	0.49	0.07	-	37.24	0.25
	Idiosyncratic	0.04 (0.20)	Post-intervention	Control	-0.08	0.06	0.23	(<0.001)	
			Pre-intervention	Heavy	0.13	0.10	0.21		
			Post-intervention	Heavy	-0.17	0.10	0.10		
			Pre-intervention	Light	-0.08	0.10	0.43		
			Post-intervention	Light	-0.06	0.10	0.58		

[a] Variance

[b] Standard deviation

[c] Standard error

[d] Highest priority critically important antimicrobial

Supplementary Table 3: Feline findings from a series of mixed effects panel regression models, measuring intervention group variation pre- and post-intervention across a range of antimicrobial prescription categories, measured as a percentage of total consultations.

Antimicrobial group	Random effects		Intervention stage	Intervention group	Fixed effects			Model fit	
	Category	Var [a] (SD [b])			Estimate	SE [c]	*P*	Chi-sq. (*P*)	R-sq.
**HPCIA [d]**	Practice	4.61 (2.15)	Pre-intervention	Control (intercept)	8.09	0.57	-	76.09	0.40
	Idiosyncratic	1.97 (1.40)	Post-intervention	Control	0.41	0.44	0.35	(<0.001)	
			Pre-intervention	Heavy	-0.04	0.81	0.97		
			Post-intervention	Heavy	-4.06	0.81	<0.0001 [e]		
			Pre-intervention	Light	-0.81	0.81	0.32		
			Post-intervention	Light	-1.76	0.81	0.03		
**Total**	Practice	7.69 (2.77)	Pre-intervention	Control (intercept)	16.51	0.76	-	11.32	0.09
	Idiosyncratic	3.89 (1.97)	Post-intervention	Control	0.92	0.62	0.14	(0.045)	
			Pre-intervention	Heavy	-1.32	1.08	0.22		
			Post-intervention	Heavy	-3.34	1.08	0.002		
			Pre-intervention	Light	-0.44	1.08	0.69		
			Post-intervention	Light	-1.76	1.08	0.10		
**Systemic**	Practice	6.30 (2.51)	Pre-intervention	Control (intercept)	13.86	0.70	-	13.40	0.11
	Idiosyncratic	3.43 (1.85)	Post-intervention	Control	0.58	0.59	0.33	(0.020)	
			Pre-intervention	Heavy	-1.19	0.99	0.23		
			Post-intervention	Heavy	-3.13	0.99	0.002		
			Pre-intervention	Light	-0.43	0.99	0.66		
			Post-intervention	Light	-1.63	0.99	0.10		
**Topical**	Practice	0.50 (0.71)	Pre-intervention	Control (intercept)	3.04	0.21	-	12.93	0.10
	Idiosyncratic	0.39 (0.62)	Post-intervention	Control	0.61	0.20	0.002	(0.024)	
			Pre-intervention	Heavy	-0.11	0.30	0.71		
			Post-intervention	Heavy	-0.53	0.30	0.07		
			Pre-intervention	Light	0.12	0.30	0.68		
			Post-intervention	Light	-0.31	0.30	0.30		
**Systemic HPCIA**	Practice	4.65 (2.16)	Pre-intervention	Control (intercept)	8.04	0.57	-	76.93	0.40
	Idiosyncratic	1.93 (1.39)	Post-intervention	Control	0.42	0.44	0.34	(<0.001)	
			Pre-intervention	Heavy	-0.08	0.81	0.92		
			Post-intervention	Heavy	-4.09	0.81	<0.0001 [f]		
			Pre-intervention	Light	-0.86	0.81	0.29		
			Post-intervention	Light	-1.81	0.81	0.03		

[a] Variance

[b] Standard deviation

[c] Standard error

[d] Highest priority critically important antimicrobial

[e] 0.0000006

[f] 0.0000005

Supplementary Table 4: Canine findings from a mixed effects panel regression model, measuring intervention group variation by month pre- (August 2018 – March 2019) and post-intervention (April – September 2019) for frequency of 'highest priority critically important antimicrobial' prescription as a percentage of total consultations. Model fit: Chi-squared value was 96.96 (*P*<0.001); R-squared value was 0.10.

Random effects		Intervention		Fixed effects		
**Category**	**Var [a] (SD [b])**	**group**	**Month**	**Estimate**	**SE [c]**	***P***
Practice	0.13 (0.36)	Control	August 2018 (intercept)	0.94	0.16	-
Idiosyncratic	0.40 (0.63)		September 2018	-0.11	0.20	0.60
			October 2018	0.04	0.20	0.83
			November 2018	-0.34	0.20	0.09
			December 2018	0.24	0.20	0.23
			January 2019	-0.30	0.20	0.13
			February 2019	-0.17	0.20	0.40
			March 2019	-0.41	0.20	0.04
			April 2019 (post-intervention)	-0.20	0.20	0.33
			May 2019	0.04	0.20	0.83
			June 2019	-0.18	0.20	0.36
			July 2019	-0.50	0.20	0.01
			August 2019	0.11	0.20	0.57
			September 2019	-0.05	0.20	0.80
			October 2019	-0.14	0.20	0.48
			November 2019	-0.22	0.20	0.27
		Heavy	August 2018	-0.11	0.23	0.62
			September 2018	-0.08	0.23	0.74
			October 2018	-0.36	0.23	0.12
			November 2018	0.06	0.23	0.78
			December 2018	-0.15	0.23	0.51
			January 2019	0.35	0.23	0.13
			February 2019	0.47	0.23	0.04
			March 2019	0.19	0.23	0.42
			April 2019 (post-intervention)	-0.28	0.23	0.22
			May 2019	-0.53	0.23	0.02
			June 2019	-0.28	0.23	0.22
			July 2019	0.20	0.23	0.40
			August 2019	-0.64	0.23	0.01
			September 2019	-0.47	0.23	0.04
			October 2019	0.03	0.23	0.91
			November 2019	-0.23	0.23	0.32
		Light	August 2018	-0.21	0.23	0.37
			September 2018	-0.19	0.23	0.42
			October 2018	-0.31	0.23	0.17
			November 2018	0.00	0.23	1.00
			December 2018	-0.11	0.23	0.64
			January 2019	0.16	0.23	0.50
			February 2019	-0.32	0.23	0.16
			March 2019	0.00	0.23	0.99
			April 2019 (post-intervention)	0.03	0.23	0.91
			May 2019	-0.22	0.23	0.34
			June 2019	-0.18	0.23	0.44
			July 2019	0.14	0.23	0.54
			August 2019	-0.13	0.23	0.57
			September 2019	-0.20	0.23	0.38
			October 2019	0.22	0.23	0.33
			November 2019	0.03	0.23	0.91

[a] Variance

[b] Standard deviation

[c] Standard error

Supplementary Table 5: Feline findings from a mixed effects panel regression model, measuring intervention group variation by month pre- (August 2018 – March 2019) and post-intervention (April – September 2019) for frequency of 'highest priority critically important antimicrobial' prescription as a percentage of total consultations. Model fit: Chi-squared value was 170.41 (*P*<0.001); R-squared value was 0.16.

Random effects		Intervention group	Month	Fixed effects		
**Category**	**Var [a] (SD [b])**			**Estimate**	**SE [c]**	***P***
Practice	5.32 (2.31)	Control	August 2018 (intercept)	8.78	0.85	-
Idiosyncratic	8.61 (2.93)		September 2018	-1.05	0.93	0.26
			October 2018	-1.14	0.93	0.22
			November 2018	-0.86	0.93	0.36
			December 2018	-1.32	0.93	0.16
			January 2019	-1.20	0.93	0.20
			February 2019	0.44	0.93	0.64
			March 2019	-1.18	0.93	0.21
			April 2019 (post-intervention)	0.08	0.93	0.93
			May 2019	-0.16	0.93	0.86
			June 2019	1.03	0.93	0.27
			July 2019	-1.17	0.93	0.21
			August 2019	0.13	0.93	0.89
			September 2019	-0.74	0.93	0.43
			October 2019	-1.48	0.93	0.11
			November 2019	-0.58	0.93	0.54
		Heavy	August 2018	-0.18	1.18	0.88
			September 2018	1.22	1.18	0.31
			October 2018	0.35	1.18	0.77
			November 2018	-1.02	1.18	0.39
			December 2018	1.02	1.18	0.40
			January 2019	-0.19	1.18	0.87
			February 2019	-1.66	1.18	0.17
			March 2019	0.68	1.18	0.57
			April 2019 (post-intervention)	-3.98	1.18	0.001
			May 2019	-3.90	1.18	0.001
			June 2019	-5.67	1.18	<0.0001 [d]
			July 2019	-3.35	1.18	0.01
			August 2019	-4.82	1.18	<0.0001 [e]
			September 2019	-3.30	1.18	0.01
			October 2019	-2.74	1.18	0.001
			November 2019	-4.06	1.18	0.001
		Light	August 2018	-1.15	1.18	0.34
			September 2018	-0.30	1.18	0.80
			October 2018	0.22	1.18	0.86
			November 2018	-0.67	1.18	0.57
			December 2018	0.58	1.18	0.63
			January 2019	-1.21	1.18	0.31
			February 2019	-2.26	1.18	0.06
			March 2019	-0.71	1.18	0.56
			April 2019 (post-intervention)	-1.18	1.18	0.32
			May 2019	-1.55	1.18	0.20
			June 2019	-2.56	1.18	0.03
			July 2019	-0.32	1.18	0.79
			August 2019	-1.92	1.18	0.11
			September 2019	-0.40	1.18	0.74
			October 2019	-1.31	1.18	0.27
			November 2019	-2.76	1.18	0.02

[a] Variance

[b] Standard deviation

[c] Standard error

[d] 0.000002

[e] 0.00004

Supplementary Table 6: Parameter estimates from six fitted mixed effects panel regression models, modelling canine and feline highest priority critically important antimicrobial prescription frequency in practices within the control, light intervention, and heavy intervention groups.

Intervention group	Random effects		Polynomial fit to month	Fixed effects			Model fit	
	Category	Var [a] (SD [b])		Estimate	SE [c]	*P*	Chi-sq.	R-sq.
DOGS								
**Control**	Practice	0.22 (0.46)	Intercept	0.80	0.11	-	26.61	0.08
	Idiosyncratic	0.44 (0.66)	x (linear)	-0.12	0.15	0.411	(0.032)	
			x^2	0.16	0.15	0.288		
			x^3	-0.07	0.15	0.636		
			x^4	-0.15	0.15	0.298		
			x^5	-0.08	0.15	0.590		
			x^6	0.02	0.15	0.902		
			x^7	-0.09	0.15	0.559		
			x^8	0.06	0.15	0.662		
			x^9	0.29	0.15	0.055		
			x^{10}	0.13	0.15	0.384		
			x^{11}	-0.40	0.15	0.007		
			x^{12}	-0.08	0.15	0.599		
			x^{13}	-0.40	0.15	0.007		
			x^{14}	0.22	0.15	0.130		
			x^{15}	-0.15	0.15	0.306		
**Light**	Practice	0.04 (0.20)	Intercept	0.72	0.05	-	36.08	0.11
	Idiosyncratic	0.24 (0.49)	x (linear)	0.13	0.11	0.238	(0.002)	
			x^2	0.15	0.11	0.167		
			x^3	0.09	0.11	0.436		
			x^4	-0.13	0.11	0.252		
			x^5	-0.19	0.11	0.085		
			x^6	0.05	0.11	0.627		
			x^7	-0.20	0.11	0.074		
			x^8	-0.14	0.11	0.219		
			x^9	0.20	0.11	0.067		
			x^{10}	-0.17	0.11	0.131		
			x^{11}	-0.43	0.11	<0.001		
			x^{12}	0.08	0.11	0.482		
			x^{13}	-0.12	0.11	0.285		
			x^{14}	0.002	0.11	0.986		
			x^{15}	-0.01	0.11	0.929		
**Heavy**	Practice	0.13 (0.36)	Intercept	0.68	0.09	-	34.71	0.10
	Idiosyncratic	0.52 (0.72)	x (linear)	-0.45	0.16	0.005	(0.003)	
			x^2	-0.08	0.16	0.610		
			x^3	0.36	0.16	0.025		
			x^4	0.11	0.16	0.490		
			x^5	-0.49	0.16	0.002		
			x^6	0.01	0.16	0.956		
			x^7	0.13	0.16	0.429		
			x^8	-0.40	0.16	0.013		
			x^9	-0.24	0.16	0.135		
			x^{10}	0.01	0.16	0.972		
			x^{11}	0.07	0.16	0.677		
			x^{12}	0.01	0.16	0.970		
			x^{13}	-0.04	0.16	0.784		
			x^{14}	0.24	0.16	0.138		
			x^{15}	-0.09	0.16	0.557		
CATS								
**Control**	Practice	7.16 (2.68)	Intercept	8.20	0.63	-	13.16	0.04
	Idiosyncratic	12.44 (3.53)	x (linear)	0.29	0.79	0.709	(0.590)	
			x^2	-0.62	0.79	0.434		
			x^3	-1.23	0.79	0.119		
			x^4	1.04	0.79	0.189		
			x^5	0.40	0.79	0.608		
			x^6	0.50	0.79	0.523		
			x^7	0.08	0.79	0.919		
			x^8	0.35	0.79	0.661		
			x^9	0.13	0.79	0.864		
			x^{10}	0.60	0.79	0.445		
			x^{11}	0.23	0.79	0.773		
			x^{12}	-1.27	0.79	0.107		
			x^{13}	-0.62	0.79	0.434		
			x^{14}	-0.25	0.79	0.749		
			x^{15}	-1.44	0.79	0.066		

**Light**	Practice	5.48 (2.34)	Intercept	7.11	0.54	-	19.71	0.06
	Idiosyncratic	7.26 (2.69)	x (linear)	-1.51	0.60	0.012	(0.184)	
			x^2	-0.67	0.60	0.269		
			x^3	-1.10	0.60	0.068		
			x^4	-0.89	0.60	0.140		
			x^5	0.09	0.60	0.887		
			x^6	0.07	0.60	0.910		
			x^7	-0.24	0.60	0.691		
			x^8	0.68	0.60	0.262		
			x^9	0.60	0.60	0.317		
			x^{10}	0.12	0.60	0.841		
			x^{11}	0.08	0.60	0.898		
			x^{12}	0.87	0.60	0.147		
			x^{13}	-0.32	0.60	0.585		
			x^{14}	0.46	0.60	0.447		
			x^{15}	-0.65	0.60	0.278		
**Heavy**	Practice		Intercept	6.23	0.43	-	180.12	0.37
	Idiosyncratic		x (linear)	-6.58	0.55	<0.001	(<0.001)	
			x^2	0.48	0.55	0.391		
			x^3	1.63	0.55	0.003		
			x^4	0.40	0.55	0.468		
			x^5	-1.51	0.55	0.006		
			x^6	-0.96	0.55	0.082		
			x^7	1.27	0.55	0.022		
			x^8	-0.18	0.55	0.746		
			x^9	-0.22	0.55	0.689		
			x^{10}	-0.13	0.55	0.811		
			x^{11}	0.46	0.55	0.406		
			x^{12}	0.94	0.55	0.090		
			x^{13}	-1.26	0.55	0.023		
			x^{14}	0.44	0.55	0.421		
			x^{15}	1.01	0.55	0.067		

[a] Variance

[b] Standard deviation

[c] Standard error

Supplementary Table 7: Canine findings from a series of mixed effects panel regression models, measuring intervention group variation pre- and post-intervention across a range of main presenting complaints for frequency of 'highest priority critically important antimicrobial' prescription, measured as a percentage of total consultations.

Main presenting complaint	Random effects		Intervention stage	Intervention group	Fixed effects			Model fit	
	Category	Var [a] (SD [b])			Estimate	SE [c]	*P*	Chi-sq. (*P*)	R-sq.
**Vaccination**	Practice	0.01 (0.06)	Pre-intervention	Control	0.12	0.03	-	18.38	0.14
	Idiosyncratic	0.02 (0.13)	Post-intervention	Control	-0.01	0.04	0.79	(0.003)	
			Pre-intervention	Heavy	-0.01	0.05	0.87		
			Post-intervention	Heavy	-0.09	0.05	0.09		
			Pre-intervention	Light	0.01	0.05	0.84		
			Post-intervention	Light	0.11	0.05	0.01		
**Other healthy**	Practice	0.28 (0.53)	Pre-intervention	Control	0.73	0.15	-	12.27	0.10
	Idiosyncratic	0.18 (0.42)	Post-intervention	Control	0.16	0.13	0.23	(0.031)	
			Pre-intervention	Heavy	-0.21	0.22	0.34		
			Post-intervention	Heavy	-0.57	0.22	0.01		
			Pre-intervention	Light	-0.34	0.22	0.11		
			Post-intervention	Light	-0.23	0.22	0.29		
**Post-operative check**	Practice	0.02 (0.15)	Pre-intervention	Control	0.43	0.13	-	2.63	0.02
	Idiosyncratic	0.31 (0.56)	Post-intervention	Control	-0.06	0.18	0.72	(0.756)	
			Pre-intervention	Heavy	0.11	0.18	0.55		
			Post-intervention	Heavy	-0.03	0.18	0.87		
			Pre-intervention	Light	0.09	0.18	0.61		
			Post-intervention	Light	0.19	0.18	0.31		
**Gastroenteric**	Practice	0.30 (0.55)	Pre-intervention	Control	0.82	0.28	-	14.83	0.12
	Idiosyncratic	1.30 (1.14)	Post-intervention	Control	-0.75	0.36	0.04	(0.011)	
			Pre-intervention	Heavy	0.50	0.40	0.21		
			Post-intervention	Heavy	0.20	0.40	0.62		
			Pre-intervention	Light	0.00	0.40	1.00		
			Post-intervention	Light	0.41	0.40	0.31		
**Respiratory**	Practice	0.00 (0.00)	Pre-intervention	Control	2.47	1.19	-	9.44	0.08
	Idiosyncratic	29.69 (5.45)	Post-intervention	Control	-0.53	1.69	0.75	(0.093)	
			Pre-intervention	Heavy	2.41	1.69	0.15		
			Post-intervention	Heavy	-1.77	1.69	0.30		
			Pre-intervention	Light	-0.80	1.69	0.64		
			Post-intervention	Light	-1.16	1.69	0.49		
**Pruritus**	Practice	2.86 (1.69)	Pre-intervention	Control	1.86	0.53	-	1.22	0.01
	Idiosyncratic	2.68 (1.64)	Post-intervention	Control	-0.10	0.52	0.84	(0.943)	
			Pre-intervention	Heavy	-0.07	0.74	0.93		
			Post-intervention	Heavy	0.22	0.74	0.76		
			Pre-intervention	Light	0.46	0.74	0.54		
			Post-intervention	Light	0.34	0.74	0.65		
**Trauma**	Practice	0.06 (0.25)	Pre-intervention	Control	0.59	0.20	-	3.46	0.03
	Idiosyncratic	0.76 (0.87)	Post-intervention	Control	-0.32	0.28	0.25	(0.629)	
			Pre-intervention	Heavy	0.19	0.29	0.50		
			Post-intervention	Heavy	0.32	0.29	0.26		
			Pre-intervention	Light	-0.08	0.29	0.77		
			Post-intervention	Light	0.34	0.29	0.23		
**Tumour**	Practice	0.00 (0.00)	Pre-intervention	Control	0.43	0.44	-	7.75	0.06
	Idiosyncratic	4.14 (2.03)	Post-intervention	Control	0.66	0.63	0.29	(0.171)	
			Pre-intervention	Heavy	0.84	0.63	0.18		
			Post-intervention	Heavy	-1.00	0.63	0.11		
			Pre-intervention	Light	0.81	0.63	0.20		
			Post-intervention	Light	-0.95	0.63	0.13		
**Kidney disease**	Practice	0.00 (0.00)	Pre-intervention	Control	6.28	2.39	-	4.08	0.04
	Idiosyncratic	15.44 (3.93)	Post-intervention	Control	-5.04	3.43	0.14	(0.537)	
			Pre-intervention	Heavy	-2.35	3.38	0.49		
			Post-intervention	Heavy	-0.05	3.34	0.99		
			Pre-intervention	Light	-5.18	3.34	0.12		
			Post-intervention	Light	-0.01	3.43	0.99		
**Other unwell**	Practice	0.66 (0.81)	Pre-intervention	Control	1.58	0.25	-	6.84	0.06
	Idiosyncratic	0.57 (0.75)	Post-intervention	Control	-0.09	0.24	0.70	(0.233)	
			Pre-intervention	Heavy	0.20	0.35	0.58		
			Post-intervention	Heavy	-0.32	0.35	0.35		
			Pre-intervention	Light	-0.13	0.35	0.72		
			Post-intervention	Light	-0.01	0.35	0.98		

[a] Variance

[b] Standard deviation

[c] Standard error

Supplementary Table 8: Feline findings from a series of mixed effects panel regression models, measuring intervention group variation pre- and post-intervention across a range of main presenting complaints for frequency of 'highest priority critically important antimicrobial' prescription, measured as a percentage of total consultations.

Main presenting	Random effects		Intervention stage	Intervention group	Fixed effects			Model fit	
	Category	Var [a] (SD [b])			Estimate	SE [c]	*P*	Chi-sq. (*P*)	R-sq.
**Vaccination**	Practice	1.89 (1.37)	Pre-intervention	Control	1.14	0.36	-	5.16	0.04
	Idiosyncratic	0.77 (0.88)	Post-intervention	Control	0.24	0.28	0.38	(0.396)	
			Pre-intervention	Heavy	-0.24	0.52	0.64		
			Post-intervention	Heavy	-0.87	0.52	0.09		
			Pre-intervention	Light	-0.04	0.52	0.94		
			Post-intervention	Light	-0.04	0.52	0.93		
**Other healthy**	Practice	12.63 (3.55)	Pre-intervention	Control	6.05	0.94	-	17.24	0.13
	Idiosyncratic	4.94 (2.22)	Post-intervention	Control	1.27	0.70	0.07	(0.004)	
			Pre-intervention	Heavy	-0.88	1.33	0.51		
			Post-intervention	Heavy	-4.26	1.33	0.001		
			Pre-intervention	Light	-1.03	1.33	0.44		
			Post-intervention	Light	-1.78	1.33	0.18		
**Post-operative check**	Practice	7.36 (2.71)	Pre-intervention	Control	4.57	0.86	-	2.65	0.02
	Idiosyncratic	7.45 (2.73)	Post-intervention	Control	-1.26	0.86	0.14	(0.754)	
			Pre-intervention	Heavy	-0.42	1.22	0.73		
			Post-intervention	Heavy	0.26	1.22	0.83		
			Pre-intervention	Light	-0.91	1.22	0.46		
			Post-intervention	Light	0.40	1.22	0.74		
**Gastroenteric**	Practice	0.00 (0.00)	Pre-intervention	Control	5.81	1.53	-	7.300	0.06
	Idiosyncratic	49.24 (7.02)	Post-intervention	Control	-1.21	2.16	0.58	(0.200)	
			Pre-intervention	Heavy	1.87	2.14	0.38		
			Post-intervention	Heavy	-2.19	2.14	0.31		
			Pre-intervention	Light	-0.98	2.14	0.65		
			Post-intervention	Light	-0.90	2.14	0.67		
**Respiratory**	Practice	97.52 (9.88)	Pre-intervention	Control	28.01	4.74	-	15.04	0.12
	Idiosyncratic	307.27 (17.53)	Post-intervention	Control	-1.71	5.84	0.64	(0.010)	
			Pre-intervention	Heavy	4.18	6.54	0.52		
			Post-intervention	Heavy	-12.18	6.54	0.06		
			Pre-intervention	Light	2.77	6.54	0.67		
			Post-intervention	Light	-2.60	6.54	0.69		
**Pruritus**	Practice	87.89 (9.38)	Pre-intervention	Control	16.56	3.06	-	13.85	0.11
	Idiosyncratic	99.89 (9.96)	Post-intervention	Control	1.97	3.15	0.53	(0.017)	
			Pre-intervention	Heavy	3.67	4.33	0.40		
			Post-intervention	Heavy	-7.95	4.33	0.07		
			Pre-intervention	Light	3.66	4.33	0.40		
			Post-intervention	Light	-4.38	4.33	0.31		
**Trauma**	Practice	85.77 (9.26)	Pre-intervention	Control	34.00	2.84	-	34.78	0.23
	Idiosyncratic	75.77 (8.69)	Post-intervention	Control	-3.97	2.75	0.15	(<0.001)	
			Pre-intervention	Heavy	-3.80	4.02	0.35		
			Post-intervention	Heavy	-13.74	4.02	0.001		
			Pre-intervention	Light	-7.08	4.02	0.08		
			Post-intervention	Light	-5.12	4.02	0.20		
**Tumour**	Practice	0.00 (0.00)	Pre-intervention	Control	13.51	3.04	-	6.25	0.06
	Idiosyncratic	195.65 (13.99)	Post-intervention	Control	2.84	4.30	0.51	(0.283)	
			Pre-intervention	Heavy	2.27	4.19	0.58		
			Post-intervention	Heavy	-6.90	4.19	0.10		
			Pre-intervention	Light	-0.70	4.19	0.87		
			Post-intervention	Light	-7.90	4.19	0.06		
**Kidney disease**	Practice	0.00 (0.00)	Pre-intervention	Control	24.37	4.40	-	11.49	0.10
	Idiosyncratic	323.39 (17.98)	Post-intervention	Control	-11.57	6.22	0.06	(0.043)	
			Pre-intervention	Heavy	-5.89	6.03	0.31		
			Post-intervention	Heavy	-4.77	6.03	0.41		
			Pre-intervention	Light	-15.38	6.03	0.01		
			Post-intervention	Light	4.86	6.03	0.42		
**Other unwell**	Practice	17.04 (4.13)	Pre-intervention	Control	15.18	1.22	-	45.52	0.29
	Idiosyncratic	12.60 (3.55)	Post-intervention	Control	-1.17	1.12	0.30	(<0.001)	
			Pre-intervention	Heavy	0.44	1.72	0.80		
			Post-intervention	Heavy	-5.49	1.72	0.001		
			Pre-intervention	Light	-1.19	1.72	0.49		
			Post-intervention	Light	-1.51	1.72	0.38		

[a] Variance
[b] Standard deviation
[c] Standard error

Supplementary Table 9: Canine and feline findings from a series of mixed effects panel regression models, measuring intervention group variation pre- and post-intervention across a range of main presenting complaints for frequency of anti-inflammatory prescription or euthanasia, or for all species, performance of cytological or bacterial culture & sensitivity diagnostic tests; all measured as a percentage of total consultations.

Category	Random effects		Intervention stage	Intervention group	Fixed effects			Model fit	
	Category	Var [a] (SD [b])			Estimate	SE [c]	*P*	Chi-sq. (*P*)	R-sq.
**Canine**									
**Anti-inflammatory**	Practice	8.52 (2.92)	Pre-intervention	Control	19.69	0.71	-	3.95	0.03
	Idiosyncratic	1.56 (1.25)	Post-intervention	Control	0.60	0.40	0.13	(0.557)	
			Pre-intervention	Heavy	1.37	1.00	0.17		
			Post-intervention	Heavy	0.43	1.00	0.67		
			Pre-intervention	Light	0.66	1.00	0.51		
			Post-intervention	Light	-0.03	1.00	0.98		
**Euthanasia**	Practice	0.08 (0.27)	Pre-intervention	Control	0.99	0.08	<0.01	5.93	0.05
	Idiosyncratic	0.05 (0.23)	Post-intervention	Control	0.08	0.07	0.29	(0.313)	
			Pre-intervention	Heavy	0.01	0.11	0.95		
			Post-intervention	Heavy	0.07	0.11	0.55		
			Pre-intervention	Light	0.09	0.11	0.41		
			Post-intervention	Light	0.07	0.11	0.54		
**Feline**									
**Anti-inflammatory**	Practice	12.68 (3.56)	Pre-intervention	Control	18.72	0.89	-	3.23	0.03
	Idiosyncratic	3.31 (1.82)	Post-intervention	Control	0.82	0.58	0.15	(0.664)	
			Pre-intervention	Heavy	-0.25	1.26	0.84		
			Post-intervention	Heavy	-0.70	1.26	0.58		
			Pre-intervention	Light	-0.65	1.26	0.61		
			Post-intervention	Light	-1.25	1.26	0.32		
**Euthanasia**	Practice	0.48 (0.70)	Pre-intervention	Control	2.59	0.20	-	6.33	0.05
	Idiosyncratic	0.29 (0.53)	Post-intervention	Control	-0.28	0.17	0.10	(0.276)	
			Pre-intervention	Heavy	-0.58	0.28	0.04		
			Post-intervention	Heavy	-0.13	0.28	0.64		
			Pre-intervention	Light	-0.17	0.28	0.55		
			Post-intervention	Light	0.11	0.28	0.68		
**All species**									
**Cytology**	Practice	4.19 (2.05)	Pre-intervention	Control	1.43	0.58	-	2.08	0.02
	Idiosyncratic	2.44 (1.56)	Post-intervention	Control	0.26	0.49	0.60	(0.839)	
			Pre-intervention	Heavy	0.60	0.81	0.46		
			Post-intervention	Heavy	0.11	0.81	0.89		
			Pre-intervention	Light	0.22	0.81	0.79		
			Post-intervention	Light	0.52	0.81	0.53		
**Bacterial culture & susceptibility**	Practice	2.30 (1.52)	Pre-intervention	Control	1.77	0.40	-	6.58	0.05
	Idiosyncratic	0.95 (0.97)	Post-intervention	Control	-0.34	0.31	0.26	(0.253)	
			Pre-intervention	Heavy	0.74	0.57	0.19		
			Post-intervention	Heavy	0.73	0.57	0.20		
			Pre-intervention	Light	-0.49	0.57	0.39		
			Post-intervention	Light	-0.05	0.57	0.94		

[a] Variance

[b] Standard deviation

[c] Standard error

Supplementary Table 10: Canine findings from a series of mixed effects panel regression models, measuring intervention group variation pre- and post-intervention across a range of antimicrobial classes, measured as a percentage of total prescriptions.

Antimicrobial class	Random effects		Intervention stage	Intervention group	Fixed effects			Model fit	
	Category	Var [a] (SD [b])			Estimate	SE [c]	*P*	Chi-sq. (*P*)	R-sq.
**Aminoglycoside**	Practice	4897.73 (69.98)	Pre-intervention	Control	102.85	19.17	-	34.12	0.23
	Idiosyncratic	2452.52 (49.52)	Post-intervention	Control	-59.05	15.67	0.0002	(<0.001)	
			Pre-intervention	Heavy	-37.80	27.11	0.16		
			Post-intervention	Heavy	-10.50	27.11	0.70		
			Pre-intervention	Light	17.55	27.11	0.52		
			Post-intervention	Light	20.75	27.11	0.44		
**Amphenicol**	Practice	3824.73 (61.84)	Pre-intervention	Control	66.20	14.53	-	8.44	0.07
	Idiosyncratic	398.79 (19.97)	Post-intervention	Control	0.15	6.31	0.98	(0.134)	
			Pre-intervention	Heavy	-22.70	20.55	0.27		
			Post-intervention	Heavy	-22.15	20.55	0.28		
			Pre-intervention	Light	-5.80	20.55	0.78		
			Post-intervention	Light	10.25	20.55	0.62		
**Beta-lactam**	Practice	4.49 (2.12)	Pre-intervention	Control	387.40	70.63	-	8.47	0.10
	Idiosyncratic	2.98 (1.73)	Post-intervention	Control	-29.85	21.01	0.16	(0.132)	
			Pre-intervention	Heavy	-111.10	99.88	0.27		
			Post-intervention	Heavy	-126.95	99.88	0.20		
			Pre-intervention	Light	105.95	99.88	0.29		
			Post-intervention	Light	112.85	99.88	0.26		
Amoxicillin	Practice	1175.57 (34.29)	Pre-intervention	Control	20.60	8.16	-	6.06	0.05
	Idiosyncratic	155.52 (12.47)	Post-intervention	Control	-5.95	3.94	0.13	(0.300)	
			Pre-intervention	Heavy	-1.60	11.54	0.89		
			Post-intervention	Heavy	-2.20	11.54	0.85		
			Pre-intervention	Light	2.25	11.54	0.85		
			Post-intervention	Light	4.70	11.54	0.68		
Clavulanic acid potentiated amoxicillin	Practice	48244.83	Pre-intervention	Control	285.50	50.46	-	9.64	0.08
	Idiosyncratic	2678.20 (51.75)	Post-intervention	Control	-21.25	16.37	0.19	(0.086)	
			Pre-intervention	Heavy	-79.80	71.36	0.26		
			Post-intervention	Heavy	-83.50	71.36	0.24		
			Pre-intervention	Light	74.95	71.36	0.29		
			Post-intervention	Light	96.05	71.36	0.18		
**1st generation cephalosporin**	Practice	5814.57 (76.25)	Pre-intervention	Control	72.95	17.95	-	11.36	0.09
	Idiosyncratic	632.18 (25.14)	Post-intervention	Control	1.15	7.06	0.88	(0.045)	
			Pre-intervention	Heavy	-28.60	25.39	0.26		
			Post-intervention	Heavy	-39.00	25.39	0.12		
			Pre-intervention	Light	28.85	25.39	0.26		
			Post-intervention	Light	9.40	25.39	0.71		
**2nd generation cephalosporin**	Practice	0.12 (0.35)	Pre-intervention	Control	0.35	0.20	-	8.90	0.07
	Idiosyncratic	0.69 (0.83)	Post-intervention	Control	-0.10	0.26	0.70	(0.113)	
			Pre-intervention	Heavy	0.45	0.28	0.11		
			Post-intervention	Heavy	-0.20	0.28	0.48		
			Pre-intervention	Light	-0.05	0.28	0.86		
			Post-intervention	Light	0.00	0.28	1.00		
**3rd generation cephalosporin**	Practice	12.14 (3.48)	Pre-intervention	Control	6.60	1.22	-	14.96	0.12
	Idiosyncratic	17.79 (4.22)	Post-intervention	Control	-2.75	1.33	0.04	(0.011)	
			Pre-intervention	Heavy	-0.45	1.73	0.79		
			Post-intervention	Heavy	-1.60	1.73	0.36		
			Pre-intervention	Light	-0.20	1.73	0.91		
			Post-intervention	Light	2.40	1.73	0.17		
**Penicillin**	Practice	0.00 (0.00)	Pre-intervention	Control	0.00	0.07	-	3.73	0.03
	Idiosyncratic	0.09 (0.30)	Post-intervention	Control	0.15	0.10	0.12	(0.589)	
			Pre-intervention	Heavy	0.05	0.10	0.60		
			Post-intervention	Heavy	-0.15	0.10	0.12		
			Pre-intervention	Light	0.00	0.10	1.00		
			Post-intervention	Light	-0.10	0.10	0.30		
**Other beta-lactams**	Practice	4.49 (2.12)	Pre-intervention	Control	1.40	0.61	-	8.47	0.07
	Idiosyncratic	2.98 (1.73)	Post-intervention	Control	-1.10	0.55	0.04	(0.132)	
			Pre-intervention	Heavy	-1.15	0.86	0.18		
			Post-intervention	Heavy	-0.30	0.86	0.73		
			Pre-intervention	Light	0.15	0.86	0.86		
			Post-intervention	Light	0.40	0.86	0.64		
**Fluoroquinolone**	Practice	535.22 (23.13)	Pre-intervention	Control	25.35	5.66	-	8.85	0.07
	Idiosyncratic	104.81 (10.24)	Post-intervention	Control	-2.25	3.24	0.05	(0.115)	
			Pre-intervention	Heavy	-8.90	8.00	0.27		
			Post-intervention	Heavy	-9.15	8.00	0.25		
			Pre-intervention	Light	8.60	8.00	0.28		
			Post-intervention	Light	13.75	8.00	0.09		
**Fusidic acid**	Practice	11805.79	Pre-intervention	Control	167.95	28.16	-	26.26	0.19

	Idiosyncratic	4054.23 (63.67)	Post-intervention	Control	-61.85	20.14	0.002	(<0.001)	
			Pre-intervention	Heavy	-36.15	39.82	0.36		
			Post-intervention	Heavy	-6.90	39.82	0.86		
			Pre-intervention	Light	52.40	39.82	0.19		
			Post-intervention	Light	50.05	39.82	0.21		
**Lincosamide**	Practice	876.42 (29.60)	Pre-intervention	Control	37.15	7.18	-	10.73	0.09
	Idiosyncratic	154.53 (12.43)	Post-intervention	Control	-5.90	3.93	0.13	(0.057)	
			Pre-intervention	Heavy	-13.65	10.15	0.18		
			Post-intervention	Heavy	-13.55	10.15	0.18		
			Pre-intervention	Light	-6.30	10.15	0.53		
			Post-intervention	Light	-8.60	10.15	0.40		
**Macrolide**	Practice	0.07 (0.26)	Pre-intervention	Control	0.30	0.13	-	5.21	0.04
	Idiosyncratic	0.28 (0.53)	Post-intervention	Control	-0.10	0.17	0.55	(0.391)	
			Pre-intervention	Heavy	-0.10	0.19	0.59		
			Post-intervention	Heavy	-0.10	0.19	0.59		
			Pre-intervention	Light	0.20	0.19	0.29		
			Post-intervention	Light	0.15	0.19	0.42		
**Nitroimidazole**	Practice	3160.27 (56.22)	Pre-intervention	Control	63.35	13.17	-	3.54	0.03
	Idiosyncratic	308.04 (17.55)	Post-intervention	Control	-3.85	5.55	0.49	(0.618)	
			Pre-intervention	Heavy	-22.50	18.62	0.23		
			Post-intervention	Heavy	-23.40	18.62	0.21		
			Pre-intervention	Light	-0.85	18.62	0.96		
			Post-intervention	Light	4.15	18.62	0.82		
**Nitroimidazole - macrolide**	Practice	23.98 (4.90)	Pre-intervention	Control	1.90	1.34	-	3.73	0.03
	Idiosyncratic	12.05 (3.47)	Post-intervention	Control	-0.35	1.10	0.75	(0.588)	
			Pre-intervention	Heavy	0.90	1.90	0.64		
			Post-intervention	Heavy	-0.45	1.90	0.81		
			Pre-intervention	Light	1.30	1.90	0.49		
			Post-intervention	Light	2.05	1.90	0.28		
**Other antimicrobials**	Practice	5509.77 (74.23)	Pre-intervention	Control	65.05	18.82	-	25.22	0.18
	Idiosyncratic	1557.00 (39.71)	Post-intervention	Control	35.65	12.56	0.004	(<0.001)	
			Pre-intervention	Heavy	-10.40	26.62	0.70		
			Post-intervention	Heavy	-30.45	26.62	0.25		
			Pre-intervention	Light	35.15	26.62	0.19		
			Post-intervention	Light	39.55	26.62	0.14		
**Sulphonamide**	Practice	0.00 (0.00)	Pre-intervention	Control	0.45	0.18	-	5.99	0.01
	Idiosyncratic	0.67 (0.82)	Post-intervention	Control	-0.45	0.25	0.07	(0.307)	
			Pre-intervention	Heavy	-0.05	0.25	0.84		
			Post-intervention	Heavy	0.30	0.25	0.23		
			Pre-intervention	Light	-0.20	0.25	0.43		
			Post-intervention	Light	0.00	0.25	1.00		
**Tetracycline**	Practice	131.07 (12.45)	Pre-intervention	Control	12.00	2.68	-	15.54	0.12
	Idiosyncratic	13.01 (3.61)	Post-intervention	Control	-3.80	1.14	0.001	(0.008)	
			Pre-intervention	Heavy	-7.55	3.80	0.05		
			Post-intervention	Heavy	-3.80	3.80	0.32		
			Pre-intervention	Light	-4.05	3.80	0.29		
			Post-intervention	Light	-1.90	3.80	0.62		

[a] Variance

[b] Standard deviation

[c] Standard error

Supplementary Table 11: Feline findings from a series of mixed effects panel regression models, measuring intervention group variation pre- and post-intervention across a range of antimicrobial classes, measured as a percentage of total prescriptions.

Antimicrobial class	Random effects		Intervention stage	Intervention group	Fixed effects			Model fit	
	Category	Var [a] (SD [b])			Estimate	SE [c]	*P*	Chi-sq. (*P*)	R-sq.
**Aminoglycoside**	Practice	50.65 (7.12)	Pre-intervention	Control	10.60	2.06	-	24.81	0.18
	Idiosyncratic	34.58 (5.88)	Post-intervention	Control	-5.40	1.86	0.004	(<0.001)	
			Pre-intervention	Heavy	-2.70	2.92	0.36		
			Post-intervention	Heavy	-0.60	2.92	0.84		
			Pre-intervention	Light	2.65	2.92	0.36		
			Post-intervention	Light	1.90	2.92	0.52		
**Amphenicol**	Practice	141.04 (11.88)	Pre-intervention	Control	8.25	2.76	-	7.60	0.06
	Idiosyncratic	11.39 (3.38)	Post-intervention	Control	1.60	1.07	0.13	(0.180)	
			Pre-intervention	Heavy	-4.10	3.90	0.29		
			Post-intervention	Heavy	-3.55	3.90	0.36		
			Pre-intervention	Light	-1.80	3.90	0.64		
			Post-intervention	Light	-3.90	3.90	0.32		
**Beta-lactam**	Practice	30885.61 (175.74)	Pre-intervention	Control	233.85	40.21	-	10.33	0.08
	Idiosyncratic	1448.65 (38.60)	Post-intervention	Control	-25.55	12.04	0.03	(0.066)	
			Pre-intervention	Heavy	-80.05	56.86	0.16		
			Post-intervention	Heavy	-61.85	56.86	0.28		
			Pre-intervention	Light	13.15	56.86	0.82		
			Post-intervention	Light	18.75	56.86	0.74		
Amoxicillin	Practice	735.33 (27.12)	Pre-intervention	Control	21.45	6.49	-	6.52	0.05
	Idiosyncratic	107.26 (10.36)	Post-intervention	Control	-7.20	3.28	0.03	(0.259)	
			Pre-intervention	Heavy	-10.85	9.18	0.24		
			Post-intervention	Heavy	0.50	9.18	0.96		
			Pre-intervention	Light	-4.50	9.18	0.62		
			Post-intervention	Light	0.80	9.18	0.93		
Clavulanic acid potentiated amoxicillin	Practice	7137.35 (84.48)	Pre-intervention	Control	89.65	20.04	-	13.97	0.11
	Idiosyncratic	895.99 (29.93)	Post-intervention	Control	-4.75	9.47	0.62	(0.016)	
			Pre-intervention	Heavy	-44.95	28.34	0.11		
			Post-intervention	Heavy	-8.05	28.34	0.78		
			Pre-intervention	Light	2.05	28.34	0.94		
			Post-intervention	Light	13.40	28.34	0.64		
**1st generation cephalosporin**	Practice	14.25 (3.78)	Pre-intervention	Control	1.70	0.97	-	3.47	0.03
	Idiosyncratic	4.40 (2.11)	Post-intervention	Control	0.10	0.67	0.88	(0.627)	
			Pre-intervention	Heavy	-0.25	1.36	0.85		
			Post-intervention	Heavy	0.50	1.36	0.71		
			Pre-intervention	Light	1.55	1.37	0.26		
			Post-intervention	Light	1.55	1.37	0.26		
**2nd generation cephalosporin**	Practice	0.03 (0.18)	Pre-intervention	Control	0.05	0.07	-	2.35	0.02
	Idiosyncratic	0.07 (0.26)	Post-intervention	Control	0.05	0.08	0.54	(0.799)	
			Pre-intervention	Heavy	-0.05	0.10	0.62		
			Post-intervention	Heavy	0.00	0.10	1.00		
			Pre-intervention	Light	0.05	0.10	0.62		
			Post-intervention	Light	-0.05	0.10	0.62		
**3rd generation cephalosporin**	Practice	6910.73 (83.13)	Pre-intervention	Control	120.85	19.91	-	29.87	0.21
	Idiosyncratic	1019.23 (31.93)	Post-intervention	Control	-13.80	10.10	0.17	(<0.001)	
			Pre-intervention	Heavy	-23.85	28.16	0.40		
			Post-intervention	Heavy	-53.60	28.16	0.06		
			Pre-intervention	Light	14.10	28.16	0.62		
			Post-intervention	Light	3.25	28.16	0.91		
**Penicillin**	Practice	0.00 (0.00)	Pre-intervention	Control	0.15	0.11	-	3.00	0.03
	Idiosyncratic	0.24 (0.49)	Post-intervention	Control	0.05	0.15	0.74	(0.700)	
			Pre-intervention	Heavy	-0.10	0.15	0.50		
			Post-intervention	Heavy	-0.20	0.15	0.18		
			Pre-intervention	Light	-0.10	0.15	0.50		
			Post-intervention	Light	-0.20	0.15	0.18		
**Fluoroquinolone**	Practice	17.78 (4.22)	Pre-intervention	Control	3.15	1.32	-	10.51	0.08
	Idiosyncratic	17.14 (4.14)	Post-intervention	Control	0.10	1.31	0.94	(0.062)	
			Pre-intervention	Heavy	3.60	1.87	0.05		
			Post-intervention	Heavy	-0.25	1.87	0.89		
			Pre-intervention	Light	2.15	1.87	0.25		
			Post-intervention	Light	0.70	1.87	0.71		
**Fusidic acid**	Practice	623.92 (24.98)	Pre-intervention	Control	32.85	5.87	-	12.41	0.10
	Idiosyncratic	65.93 (8.12)	Post-intervention	Control	-7.35	2.57	0.004	(0.030)	
			Pre-intervention	Heavy	-7.20	8.31	0.39		
			Post-intervention	Heavy	-1.25	8.31	0.88		
			Pre-intervention	Light	6.05	8.31	0.47		
			Post-intervention	Light	10.25	8.31	0.22		
**Lincosamide**	Practice	202.55 (14.23)	Pre-intervention	Control	9.80	3.35		2.77	0.02

	Idiosyncratic	22.06 (4.70)	Post-intervention	Control	-1.65	1.49	-	(0.736)	
			Pre-intervention	Heavy	-3.65	4.74	0.27		
			Post-intervention	Heavy	-1.15	4.74	0.44		
			Pre-intervention	Light	0.70	4.74	0.81		
			Post-intervention	Light	1.15	4.74	0.88		
**Macrolide**	Practice	0.00 (0.00)	Pre-intervention	Control	0.00	0.04	0.81	3.00	0.03
	Idiosyncratic	0.03 (0.16)	Post-intervention	Control	0.05	0.05	-	(0.700)	
			Pre-intervention	Heavy	0.05	0.05	0.32		
			Post-intervention	Heavy	0.00	0.05	0.32		
			Pre-intervention	Light	0.00	0.05	1.00		
			Post-intervention	Light	-0.05	0.05	1.00		
**Nitroimidazole**	Practice	4.48 (2.12)	Pre-intervention	Control	3.45	0.75	0.32	2.566	0.02
	Idiosyncratic	6.73 (2.60)	Post-intervention	Control	-0.45	0.82	-	(0.767)	
			Pre-intervention	Heavy	-0.30	1.06	0.58		
			Post-intervention	Heavy	-0.50	1.06	0.78		
			Pre-intervention	Light	-1.45	1.06	0.64		
			Post-intervention	Light	-0.70	1.06	0.17		
**Nitroimidazole - macrolide**	Practice	1.82 (1.35)	Pre-intervention	Control	1.05	0.38	0.51	2.90	0.02
	Idiosyncratic	1.00 (1.00)	Post-intervention	Control	-0.20	0.32	-	(0.716)	
			Pre-intervention	Heavy	-0.40	0.53	0.53		
			Post-intervention	Heavy	-0.50	0.53	0.45		
			Pre-intervention	Light	-0.65	0.53	0.35		
			Post-intervention	Light	-0.50	0.53	0.22		
**Other antimicrobials**	Practice	62.22 (7.89)	Pre-intervention	Control	7.35	1.98	0.35	25.34	0.18
	Idiosyncratic	16.04 (4.01)	Post-intervention	Control	4.00	1.27	-	(<0.001)	
			Pre-intervention	Heavy	-0.85	2.80	0.002		
			Post-intervention	Heavy	-3.60	2.80	0.76		
			Pre-intervention	Light	2.75	2.80	0.20		
			Post-intervention	Light	2.90	2.80	0.33		
**Tetracycline**	Practice	35.75 (5.98)	Pre-intervention	Control	4.45	1.45	0.30	4.09	0.03
	Idiosyncratic	6.56 (2.56)	Post-intervention	Control	-1.20	0.81	-	(0.537)	
			Pre-intervention	Heavy	-1.65	2.06	0.14		
			Post-intervention	Heavy	-0.15	2.06	0.42		
			Pre-intervention	Light	-0.30	2.06	0.94		
			Post-intervention	Light	-0.10	2.06	0.88		

[a] Variance

[b] Standard deviation

[c] Standard error

Supplementary Table 12: Canine antimicrobial prescription choice (percentage of total consultations)

**CANINE**	**Control group % (95% CI)** [a]		**Light intervention group % (95% CI)**		**Heavy intervention group % (95% CI)**	
**Antimicrobial class**	**Pre-intervention**	**Post-intervention**	**Pre-intervention**	**Post-intervention**	**Pre-intervention**	**Post-intervention**
Aminoglycoside	2.31 (1.91-2.71)	1.16 (0.84-1.47)	2.24 (1.58-2.90)	1.25 (0.84-1.66)	2.03 (1.50-2.55)	1.11 (0.55-1.67)
Amphenicol	1.51 (1.01-2.02)	1.62 (1.21-2.02)	1.13 (0.74-1.52)	1.44 (1.06-1.82)	1.37 (0.92-1.82)	1.37 (0.85-1.89)
Beta-lactam	7.95 (6.87-9.04)	8.02 (6.87-9.17)	8.36 (7.34-9.39)	8.19 (7.18-9.20)	7.97 (7.27-8.66)	6.66 (5.93-7.38)
Amoxicillin	*5.86 (0.64-11.09)*	*4.30 (0.01-8.58)*	*5.02 (2.43-7.60)*	*4.71 (1.96-7.46)*	*7.46 (1.71-13.22)*	*6.30 (1.92-10.69)*
Clavulanic acid potentiated amoxicillin	*71.86 (67.35-76.36)*	*71.84 (66.54-77.14)*	*71.52 (66.88-76.17)*	*75.19 (68.94-81.44)*	*73.04 (67.94-78.13)*	*77.18 (71.21-83.15)*
1st generation cephalosporin	*20.44 (16.20-24.69)*	*22.79 (18.11-27.47)*	*22.07 (17.35-26.80)*	*18.86 (13.65-24.07)*	*17.21 (14.24-20.17)*	*16.04 (12.45-19.63)*
2nd generation cephalosporin	*0.11 (0.00-0.22)*	*0.08 (0.00-0.17)*	*0.07 (0.00-0.15)*	*0.08 (0.00-0.17)*	*0.32 (0.03-0.60)*	*0.03 (0.00-0.09)*
3rd generation cephalosporin [b]	*1.90 (1.29-2.52)*	*1.27 (0.79-1.74)*	*1.45 (0.92-1.98)*	*1.42 (0.88-1.96)*	*2.46 (1.76-3.15)*	*0.92 (0.41-1.43)*
Penicillin	-	-	-	-	-	-
Other beta-lactams	*0.42 (0.00-1.00)*	*0.08 (0.00-0.21)*	*0.34 (0.00-0.68)*	-	*0.10 (0.00-0.29)*	-
Fluoroquinolone [b]	0.56 (0.43-0.68)	0.55 (0.39-0.72)	0.59 (0.46-0.72)	0.65 (0.49-0.81)	0.48 (0.28-0.69)	0.40 (0.30-0.50)
Fusidic acid	3.78 (3.37-4.20)	2.68 (2.16-3.20)	4.07 (3.72-4.42)	2.98 (2.67-3.30)	4.13 (3.71-4.54)	3.25 (2.77-3.74)
Lincosamide	0.71 (0.52-0.90)	0.75 (0.53-0.96)	0.50 (0.38-0.63)	0.40 (0.28-0.53)	0.65 (0.39-0.90)	0.46 (0.32-0.61)
Macrolide [b]	0.01 (0.00-0.01)	0.01 (0.00-0.01)	0.01 (0.00-0.02)	0.01 (0.00-0.01)	0.01 (0.00-0.01)	0.00 (0.00-0.01)
Nitroimidazole	1.44 (1.06-1.81)	1.52 (1.15-1.89)	1.16 (0.84-1.49)	1.23 (0.90-1.57)	1.29 (0.87-1.72)	1.09 (0.62-1.56)
Nitroimidazole-macrolide	0.04 (0.01-0.07)	0.04 (0.01-0.07)	0.06 (0.01-0.11)	0.06 (0.01-0.11)	0.08 (0.00-0.20)	0.04 (0.00-0.09)
Other antimicrobials	1.49 (1.11-1.88)	2.45 (2.12-2.78)	1.86 (1.45-2.27)	2.61 (2.14-3.07)	1.72 (1.53-1.91)	2.16 (1.87-2.45)
Sulphonamide	0.01 (0.00-0.02)	-	0.00 (0.00-0.01)	-	0.01 (0.00-0.03)	0.01 (0.00-0.01)
Tetracycline	0.26 (0.17-0.36)	0.20 (0.08-0.31)	0.14 (0.09-0.18)	0.11 (0.07-0.15)	0.14 (0.07-0.21)	0.14 (0.07-0.22)

[a] 95% Confidence interval

[b] Highest Priority Critically Important Antimicrobial

Supplementary Table 13: Feline antimicrobial prescription choice (percentage of total consultations)

FELINE	Control group % (95% CI) [a]		Light intervention group % (95% CI)		Heavy intervention group % (95%	
**Antimicrobial class**	**Pre-intervention**	**Post-intervention**	**Pre-intervention**	**Post-intervention**	**Pre-**	**Post-intervention**
Aminoglycoside	0.63 (0.46-0.80)	0.37 (0.18-0.56)	0.68 (0.44-0.91)	0.39 (0.22-0.55)	0.61 (0.44-	0.37 (0.23-0.51)
Amphenicol	0.47 (0.15-0.79)	0.64 (0.32-0.96)	0.33 (0.25-0.40)	0.32 (0.24-0.41)	0.32 (0.18-	0.43 (0.25-0.61)
Beta-lactam	13.01 (11.36-14.66)	12.85 (11.54-14.16)	11.52 (10.14-12.90)	11.43 (10.11-12.75)	11.43 (10.53-	10.26 (9.02-11.51)
Amoxicillin	*9.69 (2.27-17.12)*	*7.08 (1.71-12.45)*	*7.46 (2.56-12.36)*	*7.31 (2.45-12.16)*	*7.14 (3.83-*	*11.04 (6.14-15.93)*
Clavulanic acid potentiated amoxicillin	*33.63 (24.99-42.28)*	*35.23 (26.79-43.68)*	*32.44 (27.12-37.76)*	*37.43 (30.45-44.40)*	*26.28 (21.34-*	*46.99 (37.62-56.37)*
1st generation cephalosporin	*0.76 (0.45-1.08)*	*0.89 (0.38-1.39)*	*1.41 (0.37-2.45)*	*1.61 (0.52-2.70)*	*1.00 (0.21-*	*1.68 (0.25-3.11)*
2nd generation cephalosporin	*0.02 (0.00-0.06)*	*0.03 (0.00-0.10)*	*0.05 (0.00-0.14)*	*0.03 (0.00-0.10)*	-	*0.05 (0.00-0.14)*
*3rd generation cephalosporin**	*55.89 (46.54-65.25)*	*57.13 (47.51-66.74)*	*59.12 (50.88-67.36)*	*54.24 (47.78-60.69)*	*66.13 (60.68-*	*40.97 (33.04-48.89)*
Penicillin	-	-	-	-	-	-
Other beta-lactams	-	-	-	-	-	-
Fluoroquinolone*	0.18 (0.11-0.25)	0.21 (0.15-0.27)	0.25 (0.16-0.33)	0.21 (0.13-0.28)	0.48 (0.18-	0.25 (0.14-0.35)
Fusidic acid	1.95 (1.70-2.21)	1.74 (1.45-2.04)	1.95 (1.71-2.19)	1.91 (1.74-2.08)	1.98 (1.72-	1.87 (1.66-2.09)
Lincosamide	0.56 (0.31-0.81)	0.59 (0.33-0.84)	0.52 (0.23-0.81)	0.47 (0.20-0.74)	0.47 (0.30-	0.54 (0.35-0.72)
Macrolide*	-	0.00 (0.00-0.01)	-	-	0.00 (0.00-	0.01 (0.00-0.02)
Nitroimidazole	0.21 (0.10-0.32)	0.19 (0.11-0.27)	0.10 (0.06-0.14)	0.14 (0.10-0.18)	0.25 (0.15-	0.19 (0.11-0.26)
Nitroimidazole-macrolide	0.06 (0.00-0.12)	0.06 (0.02-0.11)	0.02 (0.00-0.04)	0.01 (0.00-0.03)	0.05 (0.00-	0.03 (0.00-0.06)
Other antimicrobials	0.43 (0.33-0.54)	0.74 (0.56-0.93)	0.51 (0.41-0.61)	0.74 (0.59-0.88)	0.50 (0.40-	0.59 (0.44-0.74)
Sulphonamide	-	-	0.00 (0.00-0.01)	-		-
Tetracycline	0.25 (0.08-0.42)	0.20 (0.07-0.34)	0.21 (0.14-0.28)	0.17 (0.10-0.24)	0.22 (0.05-	0.24 (0.10-0.38)

[a] 95% Confidence interval

[b] Highest Priority Critically Important Antimicrobial
