# Supplementary material for: A randomised controlled trial to reduce highest priority critically important antimicrobial prescription in companion animals
Source: Nat Commun. 2021 Mar 11;12:1593. doi: 10.1038/s41467-021-21864-3 (PMC7952375; doi:10.1038/s41467-021-21864-3)
Supplement: Supplementary file 3 — Reporting Summary [file 41467_2021_21864_MOESM3_ESM.pdf]

nature research

Corresponding author(s): David Singleton

Last updated by author(s): Nov 18, 2020

Reporting Summary

Nature Research wishes to improve the reproducibility of the work that we publish. This form provides structure for consistency and transparency in reporting. For further information on Nature Research policies, see our Editorial Policies and the Editorial Policy Checklist.

Statistics

For all statistical analyses, confirm that the following items are present in the figure legend, table legend, main text, or Methods section.

n/a	Confirmed	
☐	☒	The exact sample size (n) for each experimental group/condition, given as a discrete number and unit of measurement
☒	☐	A statement on whether measurements were taken from distinct samples or whether the same sample was measured repeatedly
☐	☒	The statistical test(s) used AND whether they are one- or two-sided<br>*Only common tests should be described solely by name; describe more complex techniques in the Methods section.*
☐	☒	A description of all covariates tested
☐	☒	A description of any assumptions or corrections, such as tests of normality and adjustment for multiple comparisons
☐	☒	A full description of the statistical parameters including central tendency (e.g. means) or other basic estimates (e.g. regression coefficient) AND variation (e.g. standard deviation) or associated estimates of uncertainty (e.g. confidence intervals)
☐	☒	For null hypothesis testing, the test statistic (e.g. F, t, r) with confidence intervals, effect sizes, degrees of freedom and P value noted<br>*Give P values as exact values whenever suitable.*
☒	☐	For Bayesian analysis, information on the choice of priors and Markov chain Monte Carlo settings
☒	☐	For hierarchical and complex designs, identification of the appropriate level for tests and full reporting of outcomes
☒	☐	Estimates of effect sizes (e.g. Cohen's d, Pearson's r), indicating how they were calculated

Our web collection on statistics for biologists contains articles on many of the points above.

Software and code

Policy information about availability of computer code

Data collection	Data was stored in a SQL database, and queried via Microsoft SQL Server Management Studio 18.
Data analysis	R version 4.0.3 was used for all statistical analyses described in this study. R packages 'randomizr' (version 0.20.), 'plm' (version 2.2-5), 'ggplot2' (version 3.3.2), and 'rcompanion' (version 2.3.26) were also used.

For manuscripts utilizing custom algorithms or software that are central to the research but not yet described in published literature, software must be made available to editors and reviewers. We strongly encourage code deposition in a community repository (e.g. GitHub). See the Nature Research guidelines for submitting code & software for further information.

Data

Policy information about availability of data

All manuscripts must include a data availability statement. This statement should provide the following information, where applicable:

- Accession codes, unique identifiers, or web links for publicly available datasets
- A list of figures that have associated raw data
- A description of any restrictions on data availability

The data that support the findings of this study are available from the corresponding author (DS) on reasonable request. The data are not publicly available due to them containing information that could compromise research participant privacy. Source data are provided with this paper.

Field-specific reporting

Life sciences study design

All studies must disclose on these points even when the disclosure is negative.

Sample size	The primary outcome was HPCIA prescription frequency post-intervention, compared to the CG. A sample size estimation indicated that to detect a 10% relative decrease in the primary outcome (standard deviation = 10%, power = 80% and $\alpha = 0.05$), 17 practices would be required in each group.
Data exclusions	No data was excluded in this study.
Replication	Regression models were replicated 10,000 times, and convergence was assessed. Convergence was successfully achieved in all cases.
Randomization	60 practices were randomly and evenly allocated into three intervention groups: the control group (CG, n practices=20, sites=40), low group (LIG, n practices=20, sites=57) or high group (HIG, n practices=20, sites=51) by simple random allocation, utilising the 'complete random allocation' function available through the 'randomizr' R package.
Blinding	As this trial involved active and varying interventions between study groups, it was not possible to blind participants to the intervention they were receiving. However, participants were unaware of which other practices were involved in the trial, nor what interventions were being performed in opposing groups. The control group received no intervention, and was not aware of their involvement in the trial.

Reporting for specific materials, systems and methods

We require information from authors about some types of materials, experimental systems and methods used in many studies. Here, indicate whether each material, system or method listed is relevant to your study. If you are not sure if a list item applies to your research, read the appropriate section before selecting a response.

Materials & experimental systems

n/a	Involved in the study	
☒	☐	Antibodies
☒	☐	Eukaryotic cell lines
☒	☐	Palaeontology and archaeology
☒	☐	Animals and other organisms
☒	☐	Human research participants
☒	☐	Clinical data
☒	☐	Dual use research of concern

Methods

n/a	Involved in the study	
☒	☐	ChIP-seq
☒	☐	Flow cytometry
☒	☐	MRI-based neuroimaging
